# Supplementary material for: A CRISPR Screen of HIV Dependency Factors Reveals That CCNT1 Is Non-Essential in T Cells but Required for HIV-1 Reactivation from Latency
Source: Viruses. 2023 Aug 31;15(9):1863. doi: 10.3390/v15091863 (PMC10535513; doi:10.3390/v15091863)
Supplement: Supplementary file 1 [file viruses-15-01863-s001.zip › FigS1.pdf]

A.

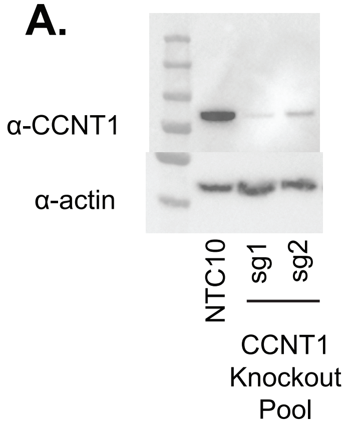

Supplemental Figure 1: Supplement to Figure 2. **A.** Above, western blot of non-targeting control (NTC10), and two different guide sequences (sg1 and sg2) targeting the *CCNT1* gene. Knockouts were performed using a lentiviral vector and are pooled knockouts of *CCNT1*. **B.** Below, sequencing chromatogram traces of wild-type and knockouts performed for each gene. Genes of interest were amplified by PCR and sent for sequencing, and analyzed by ICE analysis. Genes are labeled at the top of each chromatograph trace figure. Each line of sequence represents a CRISPR edited gene sample with the wild-type sequence (control) below. The guide sequences used are underlined in the wild-type sequence. ICE scores for each gene knockout are also available in supplemental file.

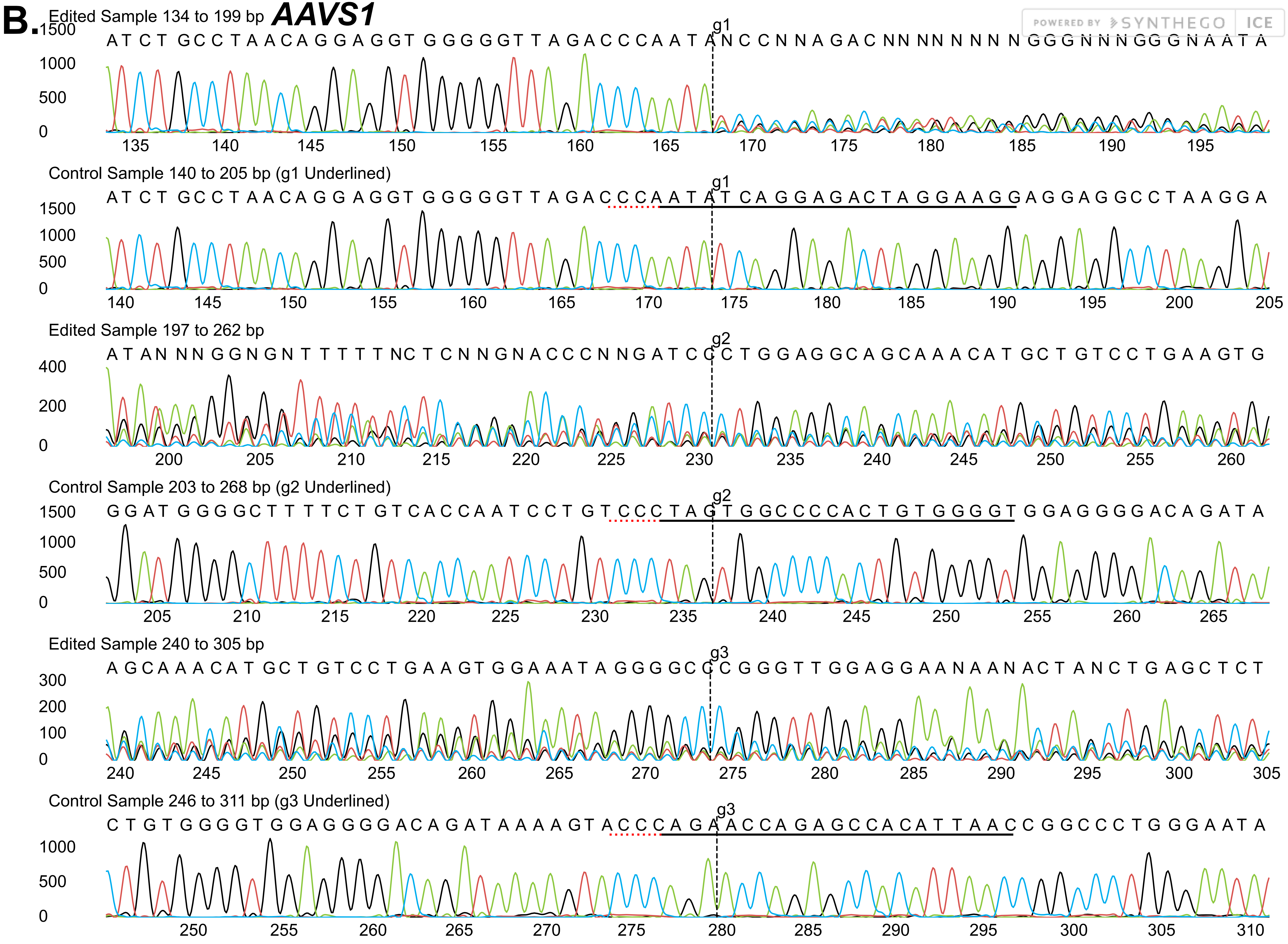

B.
AAVS1
POWERED BY SYNTHEGO ICE
Edited Sample 134 to 199 bp
g1
Control Sample 140 to 205 bp (g1 Underlined)
g1
Edited Sample 197 to 262 bp
g2
Control Sample 203 to 268 bp (g2 Underlined)
g2
Edited Sample 240 to 305 bp
g3
Control Sample 246 to 311 bp (g3 Underlined)
g3

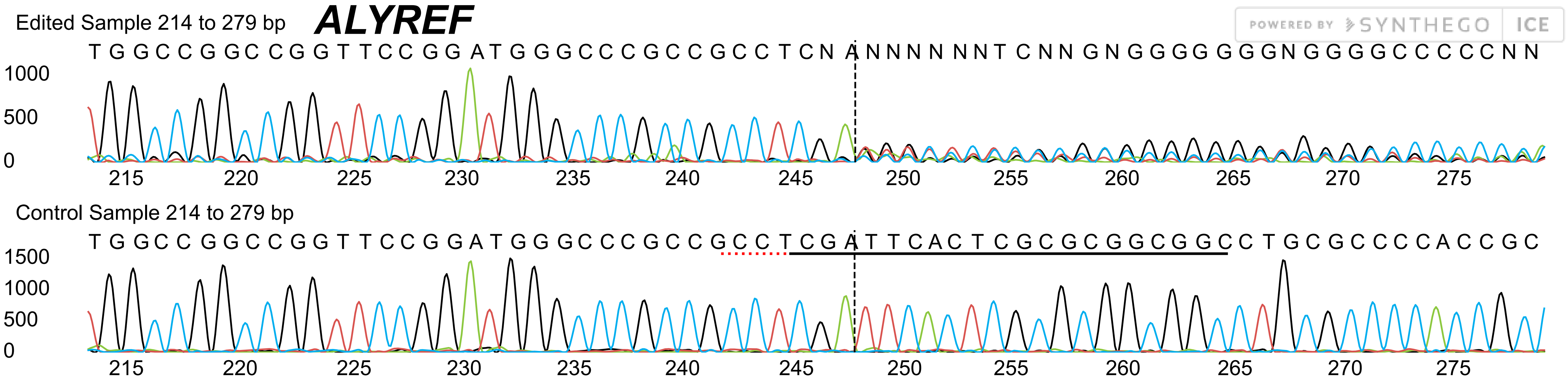

Edited Sample 214 to 279 bp
ALYREF
POWERED BY SYNTHEGO ICE
Control Sample 214 to 279 bp

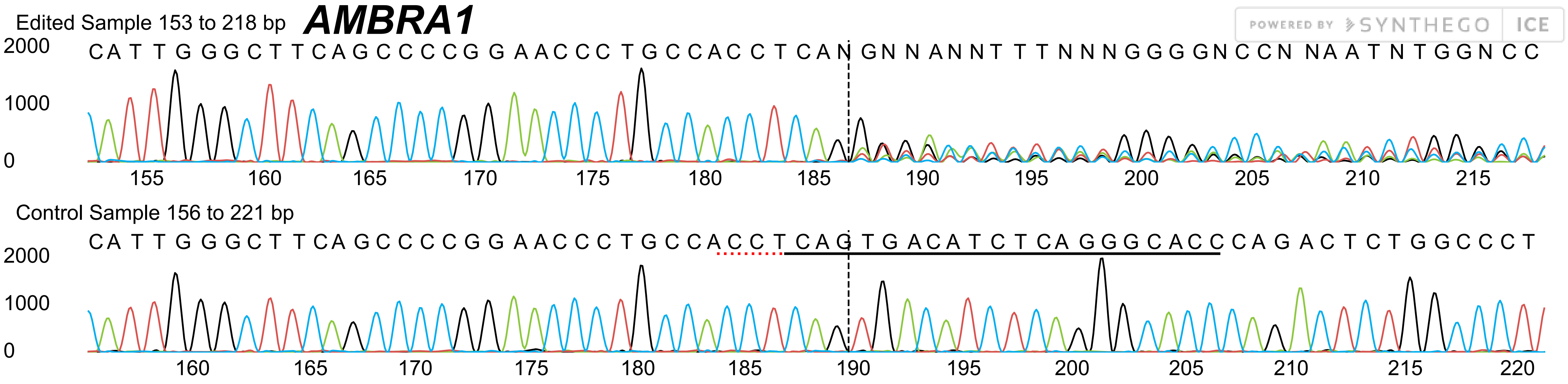

Edited Sample 153 to 218 bp
AMBRA1
POWERED BY SYNTHEGO ICE
CATTGGGCTTCAGCCCCGGAACCCTGCCACCTCANGNNANNTTNNNGGGGNCCNNAATNTGGNCC
Control Sample 156 to 221 bp
CATTGGGCTTCAGCCCCGGAACCCTGCCACCTCAGTGACATCTCAGGGCACCCAGACTCTGGCCCT

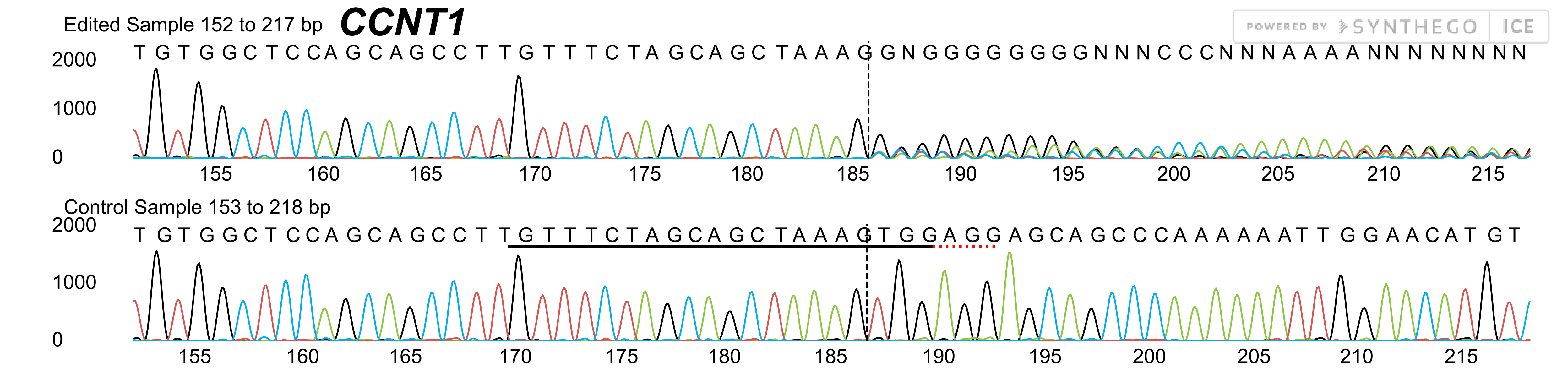

Edited Sample 152 to 217 bp
CCNT1
POWERED BY SYNTHEGO ICE
T G T G G C T C C A G C A G C C T T G T T T C T A G C A G C T A A A G G N G G G G G G G N N N C C C N N N A A A A N N N N N N N N
2000
1000
0
155 160 165 170 175 180 185 190 195 200 205 210 215
Control Sample 153 to 218 bp
2000
T G T G G C T C C A G C A G C C T T G T T T C T A G C A G C T A A A G T G G A G G A G C A G C C C A A A A A A T T G G A A C A T G T
1000
0
155 160 165 170 175 180 185 190 195 200 205 210 215

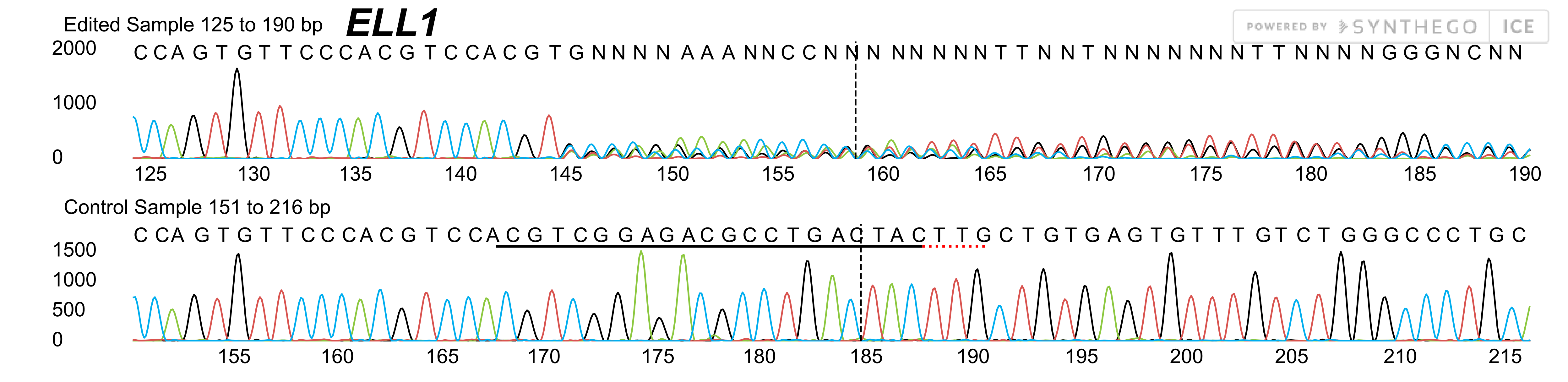
Edited Sample 125 to 190 bp
ELL1
POWERED BY SYNTHEGO ICE
CCAGTGTTCCCACGTCCACGTGNNNNAAANNCCNNNNNNNNTTNNTNNNNNNNTTNNNNGGGNCNN
2000
1000
0
125 130 135 140 145 150 155 160 165 170 175 180 185 190
Control Sample 151 to 216 bp
CCAGTGTTCCCACGTCCACGTCGGAGACGCCTGACTACTTGCTGTGAGTGTTTGTCTGGGCCCTGC
1500
1000
500
0
155 160 165 170 175 180 185 190 195 200 205 210 215

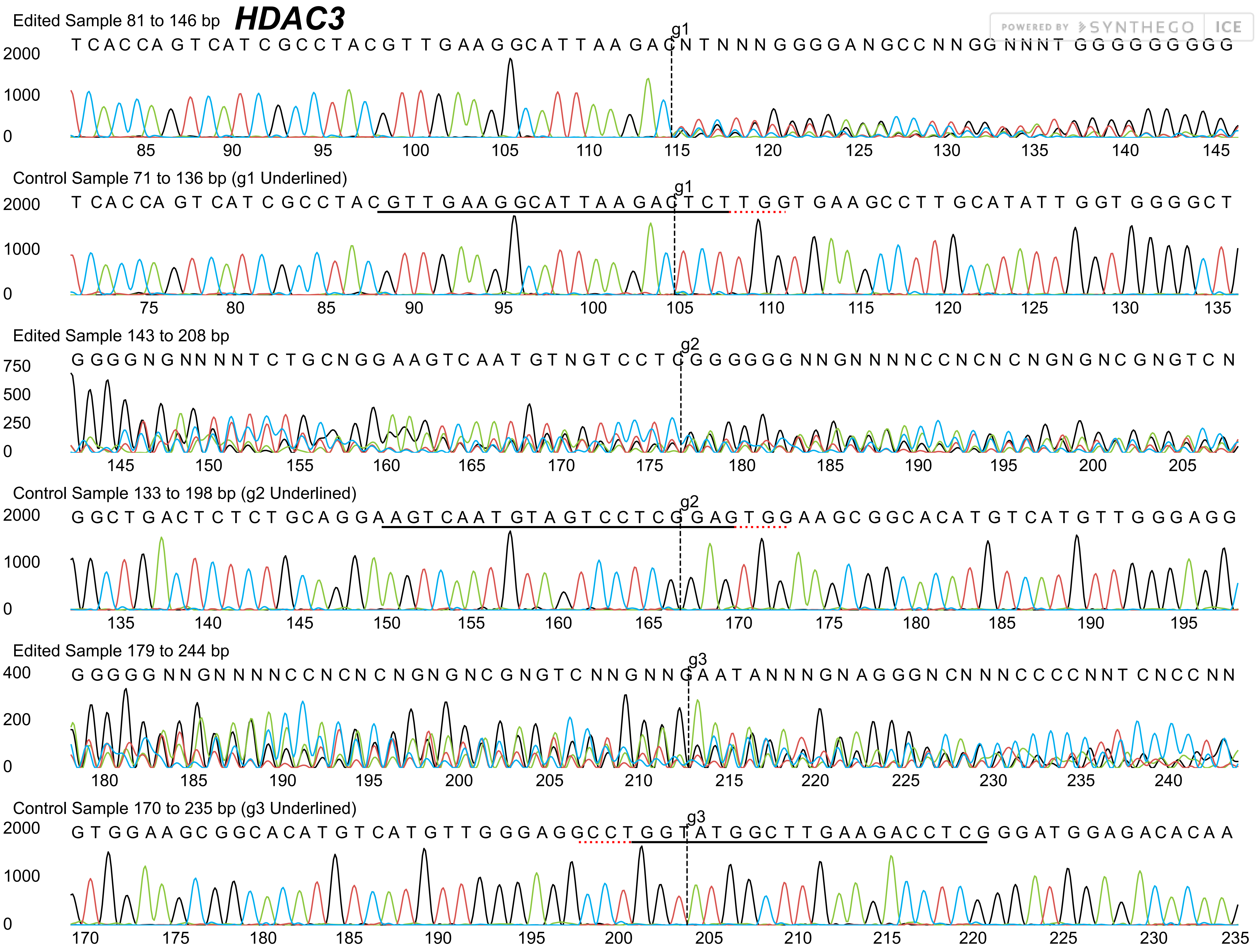

HDAC3
POWERED BY SYNTHEGO ICE
Edited Sample 81 to 146 bp
g1
TCACCAGTCATCGCCTACGTTGAAGGCATTAAGACNTNNNGGGANGCCNNGGNNNTGGGGGGGG
Control Sample 71 to 136 bp (g1 Underlined)
g1
TCACCAGTCATCGCCTACGTTGAAGGCATTAAGACTCTTGGTGAAGCCTTGCATATTGGTGGGGCT
Edited Sample 143 to 208 bp
g2
GGGGNGNNNNTCTGCNGGAAGTCAATGTNGTCCTCGGGGGNNGNNNNCCNCNCNGNGNCGNGTCN
Control Sample 133 to 198 bp (g2 Underlined)
g2
GGCTGACTCTCTGCAGGAAGTCAATGTAGTCCTCGGAGTGGAAGCGGCACATGTCATGTTGGGAGG
Edited Sample 179 to 244 bp
g3
GGGGGNNGNNNNCCNCNCNGNGNCGNGTCNNGNNGAATANNNGNAGGGNCNNNCCCCNNTCNCCNN
Control Sample 170 to 235 bp (g3 Underlined)
g3
GTGGAAGCGGCACATGTCATGTTGGGAGGCCTGGTATGGCTTGAAGACCTCGGGATGGAGACACAA

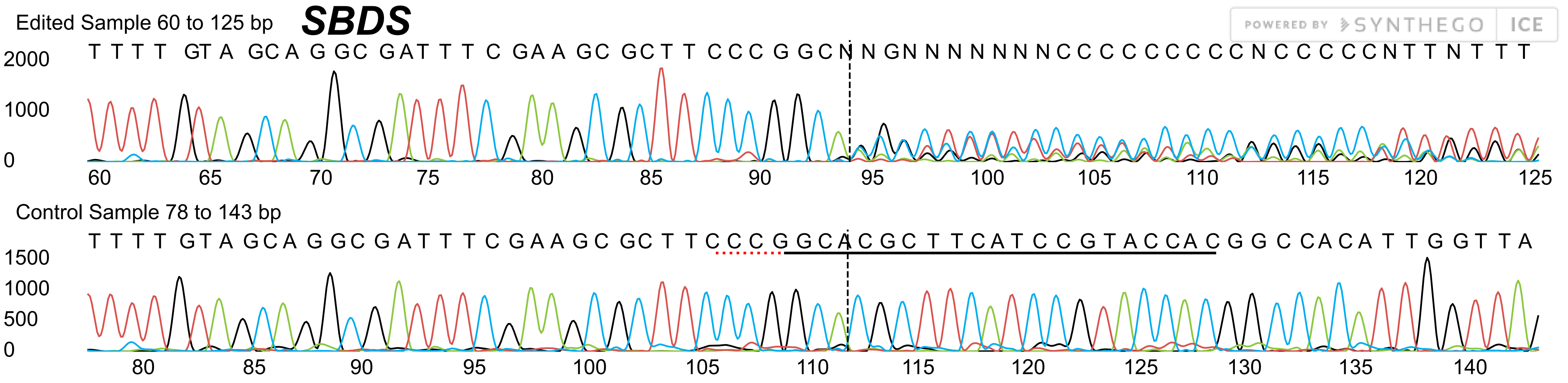

SBDS
POWERED BY SYNTHEGO ICE
Edited Sample 60 to 125 bp
Control Sample 78 to 143 bp

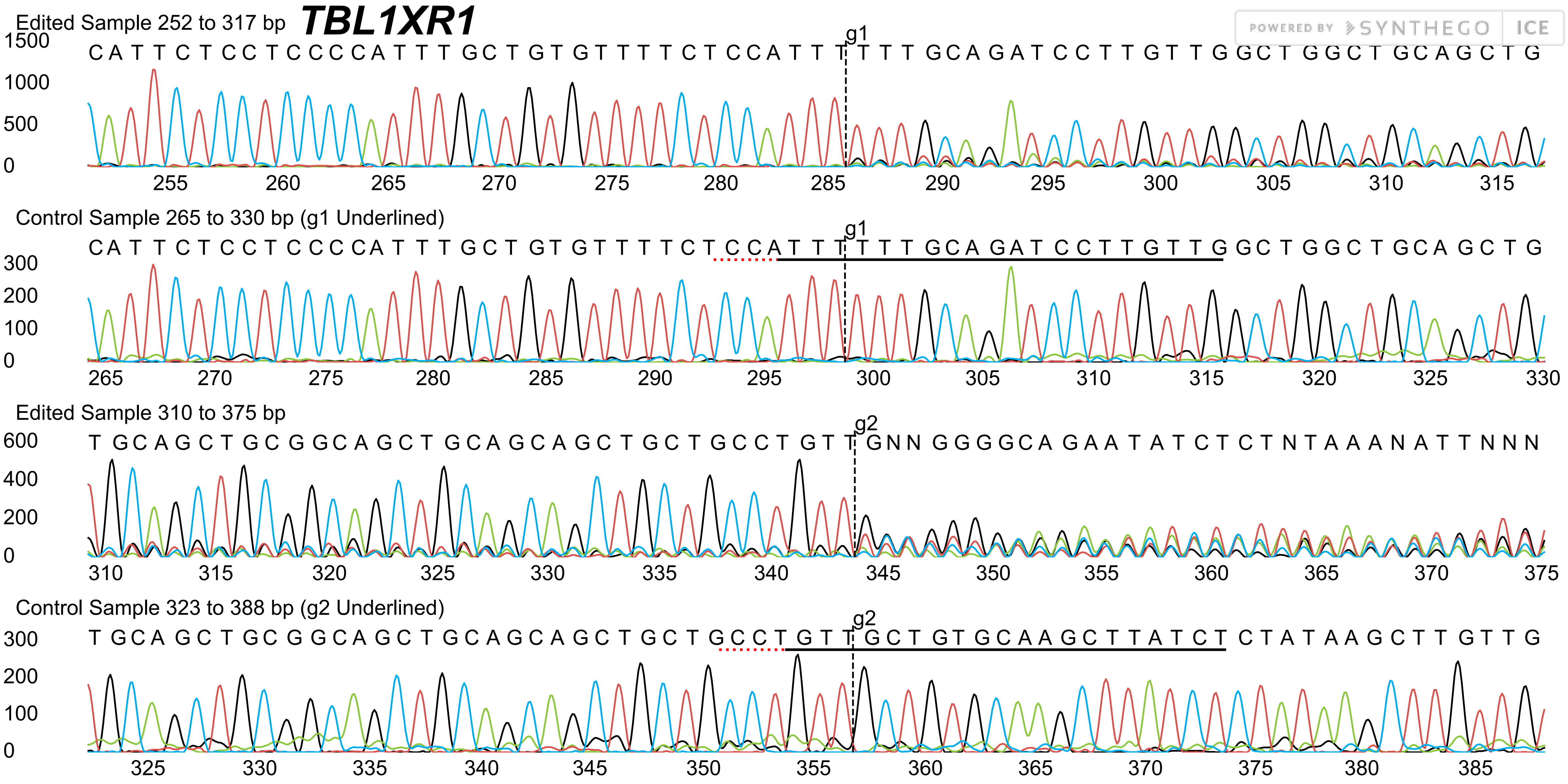

TBL1XR1
POWERED BY SYNTHEGO ICE
Edited Sample 252 to 317 bp
Control Sample 265 to 330 bp (g1 Underlined)
Edited Sample 310 to 375 bp
Control Sample 323 to 388 bp (g2 Underlined)
g1
g2

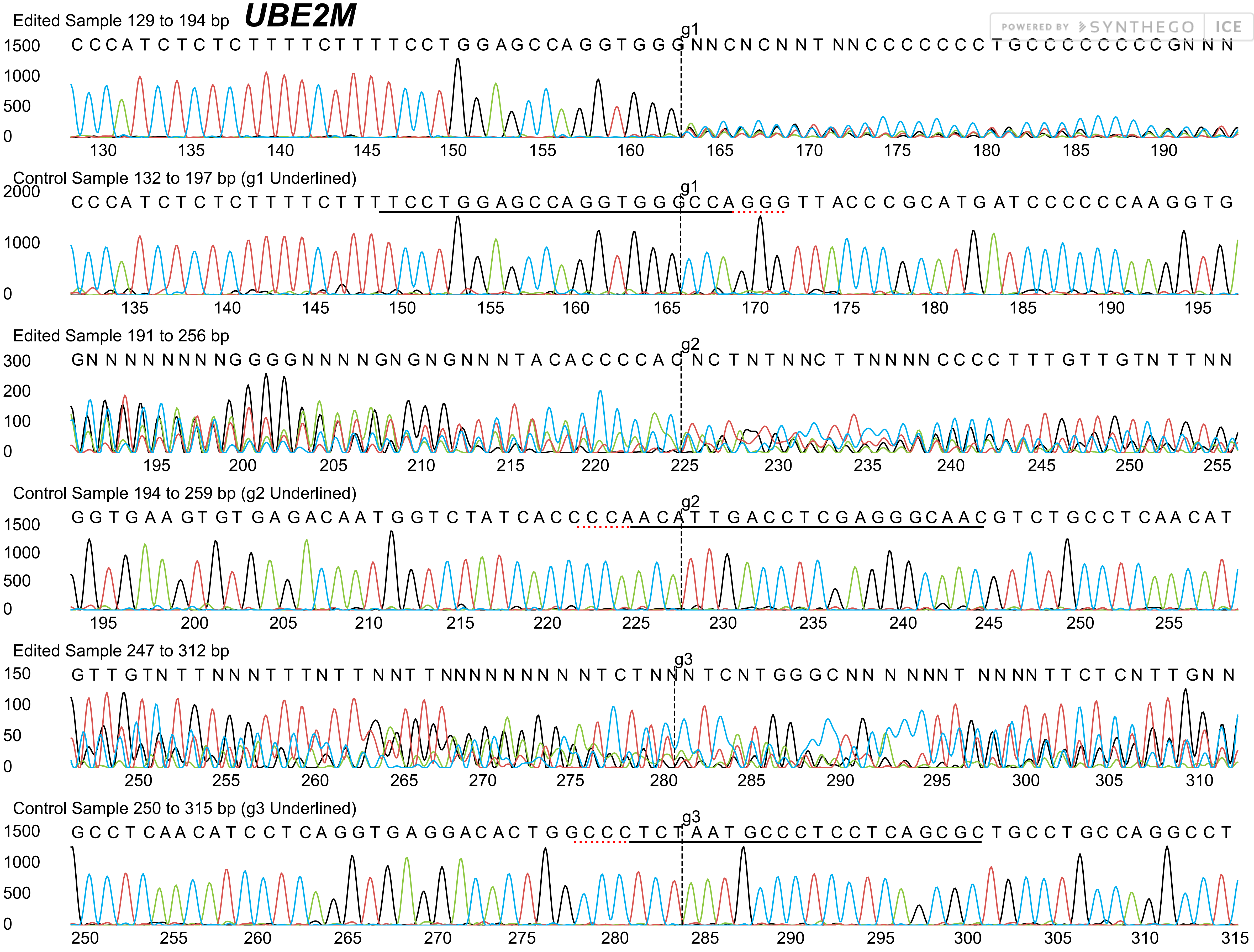

UBE2M
POWERED BY SYNTHEGO ICE
Edited Sample 129 to 194 bp
g1
Control Sample 132 to 197 bp (g1 Underlined)
Edited Sample 191 to 256 bp
g2
Control Sample 194 to 259 bp (g2 Underlined)
Edited Sample 247 to 312 bp
g3
Control Sample 250 to 315 bp (g3 Underlined)
