# Supplementary material for: A CRISPR Screen of HIV Dependency Factors Reveals That CCNT1 Is Non-Essential in T Cells but Required for HIV-1 Reactivation from Latency
Source: Viruses. 2023 Aug 31;15(9):1863. doi: 10.3390/v15091863 (PMC10535513; doi:10.3390/v15091863)
Supplement: Supplementary file 1 [file viruses-15-01863-s001.zip › FigS2.pdf]

A.

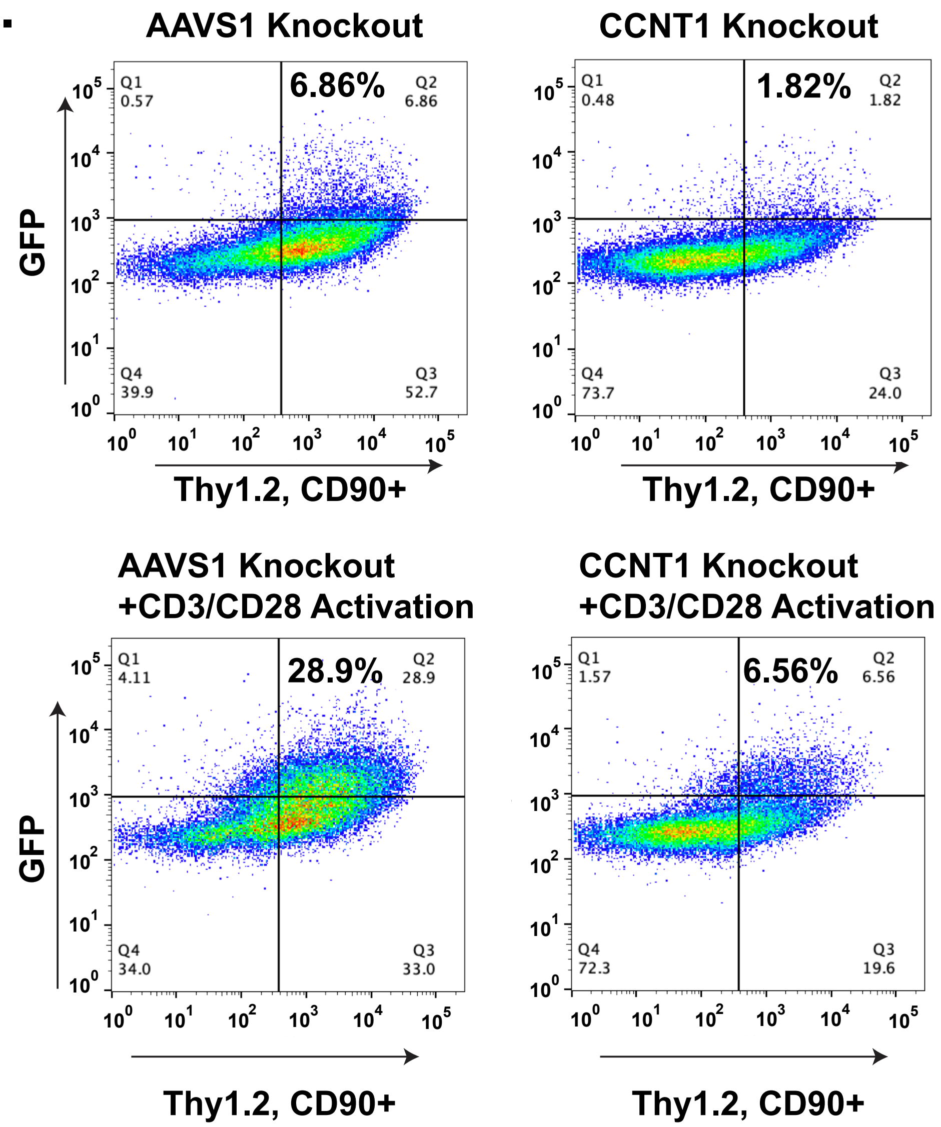

Supplemental Figure S2: Representative flow gating of primary latency model. Flow plots are shown from one representative donor. CD4+ T Cells infected with dual-reporter HIV virus, gene knockouts performed, latency was established, and treated with anti-CD3/anti-CD28 antibodies for 24 hours (see Methods) to assess reactivation potential with T cell receptor co-stimulation.
